# Anti-angiogenic effects of pterogynidine alkaloid isolated from *Alchornea glandulosa*

**DOI:** 10.1186/1472-6882-9-15

**Published:** 2009-05-22

**Authors:** Flávia CM Lopes, Ana Rocha, Ana Pirraco, Luis O Regasini, Dulce HS Silva, Vanderlan S Bolzani, Isabel Azevedo, Iracilda Z Carlos, Raquel Soares

**Affiliations:** 1 Department of Clinical Analysis, Faculty of Pharmaceutical Sciences of Araraquara, São Paulo State University – UNESP, R. Expedicionários do Brasil, 1621, CEP: 14801-902, Araraquara, São Paulo, Brazil; 2 Department of Biochemistry (U38/FCT), Faculty of Medicine, University of Porto, Portugal; 3 Department of Organic Chemistry, Araraquara Institute of Chemistry, São Paulo State University – UNESP, Araraquara, São Paulo, Brazil

## Abstract

**Background:**

Angiogenesis, a complex multistep process that comprehends proliferation, migration and anastomosis of endothelial cells (EC), has a major role in the development of pathologic conditions such as inflammatory diseases, tumor growth and metastasis. Brazilian flora, the most diverse in the world, is an interesting spot to prospect for new chemical leads, being an important source of new anticancer drugs. Plant-derived alkaloids have traditionally been of interest due to their pronounced physiological activities. We investigated the anti-angiogenic potential of the naturally occurring guanidine alkaloid pterogynidine (Pt) isolated from the Brazilian plant *Alchornea glandulosa*. The purpose of this study was to examine which features of the angiogenic process could be disturbed by Pt.

**Methods:**

Human umbilical vein endothelial cells (HUVEC) were incubated with 8 μM Pt and cell viability, proliferation, apoptosis, invasion and capillary-like structures formation were addressed. Nuclear factor κB (NFκB), a transcription factor implicated in these processes, was also evaluated in HUVEC incubated with Pt. Quantifications were expressed as mean ± SD of five independent experiments and one-way analysis of variance (ANOVA) followed by the Dunnet test was used.

**Results:**

A significant decrease in proliferation and invasion capacity and an effective increase in apoptosis as assessed by bromodeoxyuridine (BrdU), double-chamber and terminal transferase dUTP nick end labeling (TUNEL) assay, respectively, have been found. Pt also led to a drastic reduction in the number of capillary-like structures formation when HUVEC were cultured on growth factor reduced-Matrigel (GFR-Matrigel) coated plates. In addition, incubation of HUVEC with Pt resulted in reduced NFκB activity.

**Conclusion:**

These findings emphasize the potential use of Pt against pathological situations where angiogenesis is stimulated as tumor development.

## Background

Angiogenesis, the formation of new capillaries from preexisting vessels, plays a major role in several physiological and pathological events. It is an important process during pathologic conditions such as inflammatory diseases, tumor growth and metastasis [1]. This complex multistep process comprehends the extracellular matrix degradation, endothelial cells (EC) proliferation, migration and anastomosis, ending up by the recruitment and adhesion of pericytes or smooth muscle cells [2].

In tumor pathogenesis, angiogenesis is crucial and it sustains malignant cells with nutrients and oxygen. Tumor cells secrete various growth factors which triggers EC to form new capillaries. Preventing the expansion of new blood vessel networks results in reduced tumor size and metastases [3]. Since angiogenesis is essential for tumor development and tumor vasculature is considered an optimal target for anti-cancer strategies, many researchers are testing compounds of different origin and mechanism of action trying to develop antiangiogenic agents as a treatment for malignancy or as an adjunct to standard chemotherapeutic regimens [4-7].

Throughout history, natural products have afforded a rich source of compounds that have found many applications in the fields of medicine, pharmacy and biology. Within the sphere of cancer, a number of important new commercialized drugs have been obtained from natural sources, by structural modification of natural compounds, or by the synthesis of new compounds, designed following a natural compound as model [8].

The Brazilian flora, the most diverse in the world, is an interesting spot to prospect for new chemical leads due to its species diversity and associated chemical richness [9]. Therefore, plants from the Brazilian forests are important sources of new anticancer drugs [9-11].

Among several types of compounds obtained from plants, alkaloids have traditionally been of interest due to their pronounced and various physiological activities in animals and humans [12]. The most famous examples of anticancer alkaloids are taxol (clinically available since 1994) from the western yew, *Taxus brevifolia*, and camptothecin and derivatives, currently in clinical trials, from *Camptotheca acuminata *[13-15]. The alkaloid taspine hydrochloride founded in Sangre de grado plant is also considered a potential anticancer agent [16] and homoharringtonine, an alkaloid isolated from the Chinese tree *Cephalotaxus harringtonia *(Cephalotaxacea), has shown efficacy against various leukemias [17].

In the plant kingdom, guanidine alkaloids are restricted to the families Euphorbiaceae and Leguminosae [18]. *Alchornea glandulosa *Poepp & Endl. (Euphorbiaceae) is a plant distributed from southeast to south of Brazil, mainly in the Atlantic pluvial Forest [19]. Popularly known as Tapiá, it was found to exert anti-inflammatory activity and have therapeutic potential in the control of inflammatory disorders [20].

**Figure 1:**
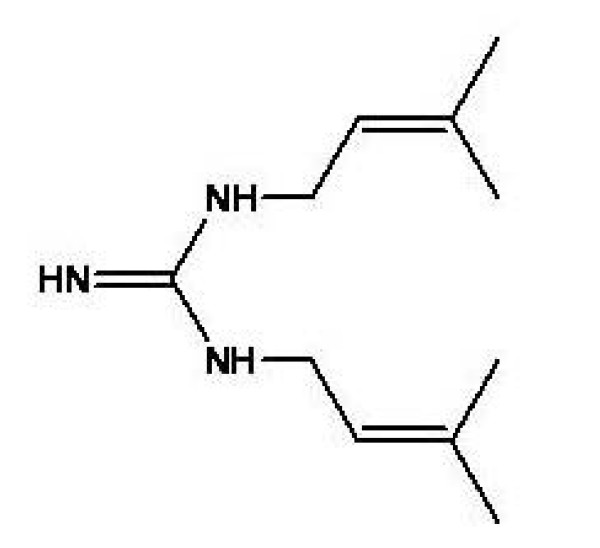
**Pterogynidine (Pt)**. Molecular structure of the guanidine alkaloid pterogynidine.

Pterogynidine (Pt) (Figure 1), a naturally occurring guanidine alkaloid, was isolated from the leaves of *A. glandulosa*. In a previous report, Bolzani et al. [18] tested the cytotoxic activity of Pt in genetically engineered strains of the yeast *Saccharomyces cerevisiae *for mechanism-based anticancer activity. This alkaloid was found to be active in the mutant strain RS 321, suggesting its potential as antitumoral agent.

However, the potential effect of Pt on angiogenesis had not yet been studied. The aim of the current research was to identify the effects of Pt within the angiogenic process. Accordingly, the present study addressed cell viability, proliferation, apoptosis, invasion and capillary-like structures formation in EC cultures. Moreover, knowing that NFκB signaling pathway is involved in all these cell processes, we investigated if the mechanism of Pt antiangiogenic activity relies on this transcription factor NFκB signaling pathway.

## Methods

### Phytochemical Procedures

Leaves of *Alchornea glandulosa *Poepp & Endl. (Euphorbiaceae) were collected in the Biological Reserve and Experimental Station at Mogi Guaçu, São Paulo State, Brazil, in March 2005, and a voucher specimen (SP319257) has been deposited in the herbarium of the Botanic Institute (São Paulo-SP, Brazil). The shade-dried leaves (1.5 kg) were ground and defatted with *n*-hexane (3.5 L × 3, at room temperature) and exhaustively extracted by maceration with methanol (4.2 L × 3). The methanol extract was concentrated under reduced pressure to yield 3.8 g of a syrup residue. The concentrate was then diluted with methanol:water (4:1) and successively partitioned with ethyl acetate and *n*-butanol. After solvent removal using rotaevaporator, each partition phase yield 2.5 g and 0.8 g, respectively. The ethyl acetate residue (2.0 g) was chromatographed by gel permeation over Sephadex LH-20 (Pharmacia^®^) eluted with methanol to afford eleven fractions (A1-A11). Chromatographic purification of fraction A3 (330 mg) by RP-HPLC [acetonitrile:water:acetic acid (87:12.5:0.5), UV detection at 237 nm; flow rate 12 mL/min] led to the isolation of Pt (183 mg). The identification was based on analysis of ^1^H, ^13^C NMR and MS data, as well as by comparison with authentic material available in our laboratory [18].

### Culture of Human Umbilical Vein Endothelial Cells (HUVEC)

Human umbilical vein endothelial cells (HUVEC) were obtained from ScienceCell Research Labs (San Diego, USA), is a primary endothelial cell culture most commonly used for angiogenic assays. Cells were used between passages 5 and 15. HUVEC cultures were first established in M199 medium (Sigma-Aldrich, Portugal) supplemented with 20% fetal bovine serum (FBS) (Invitrogen Life Technologies, Scotland, UK), 1% penicillin/streptomycin (Invitrogen Life Technologies, Scotland, UK), 0.01% heparin (Sigma-Aldrich Portugal) and 30 μg/ml endothelial cell growth supplement (ECGS) (Sigma-Aldrich, Portugal), and maintained at 37°C in a humidified 5% CO_2 _atmosphere. Cells were seeded on plates coated with 0.2% gelatin (Sigma) and allowed to grow. Cell media were changed every other day.

### Preparation of Pt samples

Pterogynidine (Pt) was dissolved in dimethyl sulfoxide (DMSO) and added to cell culture M199 medium at a concentration range of 8 μM to 128 μM for MTT assay purposes. The concentration of 8 μM was established for the following experiments according to MTT findings. Every experiment was carried out by adding Pt to serum-free M199 medium containing ECGS during 24 h as established in previous assays (unpublished data). Control cells were incubated with M199 medium and 0.1% DMSO (vehicle). DMSO concentration was 0.1% in every culture.

### MTT Assay

HUVEC (5 × 10^4^) were allowed to grow until 70–90% confluence and then incubated with Pt at a concentration range of 8, 16, 32, 64 and 128 μM or 0.1% DMSO M199 medium (control) for 24 h. After the incubation period, cells were washed twice with PBS and subjected to MTT [3-(4,5-dimethylthiazol-2-yl)-2,5-diphenyl tetrazolium bromide] assay, as previously described [21]. The choice of the indicated concentrations of Pt in the MTT assay was based on previous research of our group (unpublished data).

### BrdU Proliferation Assay

HUVEC (5 × 10^4^) were cultured following standard conditions or the treatment procedures for 24 h. Cells were then washed twice with PBS and subjected to *in situ *detection with BrdU (bromodeoxyuridine), an index of DNA synthesis and cell proliferation. Cells were incubated with BrdU solution at a final concentration of 0.01 mM for 24 h and then the *in situ *detection was performed using BrdU In situ Detection Kit (BD Biosciences Pharmingen, USA), according to the manufacturer's instructions. The results are given as mean ± SD and expressed as percentage of proliferating cells. The percentage of proliferating cells was evaluated at a 200× magnification field. A total of 1,000 nuclei were evaluated. Five independent experiments were performed.

### Tunel Assay

Cells (2 × 10^4^) plated in glass coverslips were grown for 24 h and then incubated with Pt or 0.1% DMSO M199 medium (control) for 24 h. TUNEL assay (Terminal deoxynucleotidyl transferase mediated deoxyuridine triphosphate nick-end labeling) was performed using the In Situ Cell Death Detection kit (Roche Diagnostics, Basel, Switzerland), according to the manufacturing instructions [21,22]. Apoptosis was determined as the percentage of positive cells per 1000 DAPI-stained nuclei. Glass coverslips were visualized under a fluorescence microscope (Nikon Eclipse 50i) at a magnification of 200×.

### Invasion Assay

The invasive cell behavior in the presence of Pt was quantified *in vitro *using a double-chamber assay by counting the number of cells that invaded Transwell BD-Matrigel basement membrane matrix inserts (BD-Biosciences, Belgium), according to manufacturer's instructions. FBS was used as chemoattractant. Results represent the ratio between invading cells in Pt treated cultures and in control cultures for the same initial amount of cells seeded.

### Matrigel assay – Tube Formation Index

Matrigel assay was performed on growth factor reduced-Matrigel (GFR-Matrigel) (BD Biosciences, Belgium)-coated plates for 24 h as previously described [23]. Briefly, cells (2 × 10^4^) were cultured on GFR-Matrigel-coated plates for 24 h, in medium containing 8 μM Pt or 0.1% DMSO M199 medium (control). When cultured on Matrigel, EC assemble into capillary-like structures. The number of cord-like structures was then measured and photomicrographs were also taken using an inverted microscope (400× magnification). Each cord portion between the ramifications was considered one cord unit. Mean values were obtained by evaluating the whole cultures of each well under the same treatment. A semi-quantitative measurement of cord formation in GFR-Matrigel cultured HUVEC was developed (tube formation index) as previously described [23].

### NFκB activity

NFκB activity was determined by ELISA assay. As previously reported [24], unstimulated whole cell lysates were obtained and quantified by BCA protein assay kit (Pierce, USA). NFκB activity in cell lysates obtained from 8 μM Pt or control (DMSO treated) HUVEC was measured using TransAM NFκB p50 transcription factor assay kit (Active Motif, CA, USA). In brief, protein samples (20 μg) were added to a 96-well plate with immobilized oligonucleotide containing the NFκB consensus site. Sample wells were incubated with NFκB p50 subunit primary antibody, followed by incubation with HRP-conjugated secondary antibody. Quantification was performed at 450 nm and 650 nm using a plate reader (Thermo Electron Corporation, Multiskan Ascent, USA).

### Statistical Analysis

All experiments were performed in triplicate. Quantifications are defined as mean ± SD of five independent experiments and expressed as percentage of control, which was considered to be 100%. Statistical significance of difference between various groups was evaluated by one-way analysis of variance (ANOVA test) followed by the Tukey-Kramer multiple comparisons test. For comparison between two groups, one-way analysis of variance (ANOVA test) followed by the Dunnet test was used. A difference between experimental groups was considered statistically significant whenever the p value was < 0.05.

## Results

### Effects of Pt in HUVEC

**Figure 2:**
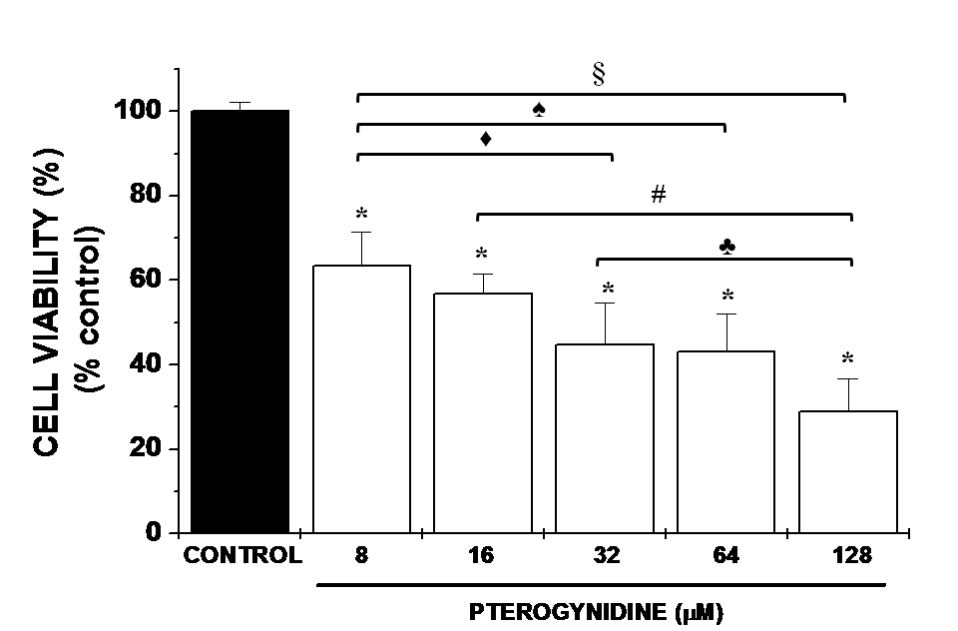
**Effect of Pt in HUVEC viability evaluated by MTT assay**. After incubation with 8 μM Pt, the percentage of cell viability was 63.33% ± 8.12% as evaluated by MTT assay. Results are expressed as percentage of control cells. Bars represent mean ± SD. Assays were repeated five times and performed in triplicate. Statistical significance between various groups was evaluated by one-way analysis of variance (ANOVA test) followed by the Tukey-Kramer multiple comparisons test. *p < 0.001 vs. Control; black diamond: p < 0.01 of 8 μM vs. 32 μM; black spade: p < 0.01 of 8 μM vs. 64 μM; §p < 0.001 of 8 μM vs. 128 μM; #p < 0.001 of 16 μM vs. 128 μM; black club: p < 0.05 of 32 μM vs. 128 μM.

To study whether Pt exerted any effect on EC, HUVEC were incubated with five different Pt concentrations (8, 16, 32, 64 and 128 μM) and cell viability was assessed by MTT test. Incubation with Pt resulted in decreased cell viability in a dose-dependent manner as evaluated by MTT assay. A reduction to 63% of control values was found upon treatment with 8 μM Pt (63.33% ± 8.12%; *p < 0.01 vs. control) (Figure 2). In addition, cell viability of 8 μM Pt was also significantly different from the three highest Pt concentrations. Incubation of HUVEC with the highest concentration of Pt (128 μM) effectively decreased cell viability even when compared to 8 μM, 16 μM and 32 μM Pt. These findings prompted us to use the 8 μM concentration of the alkaloid in the following experiments.

**Figure 3:**
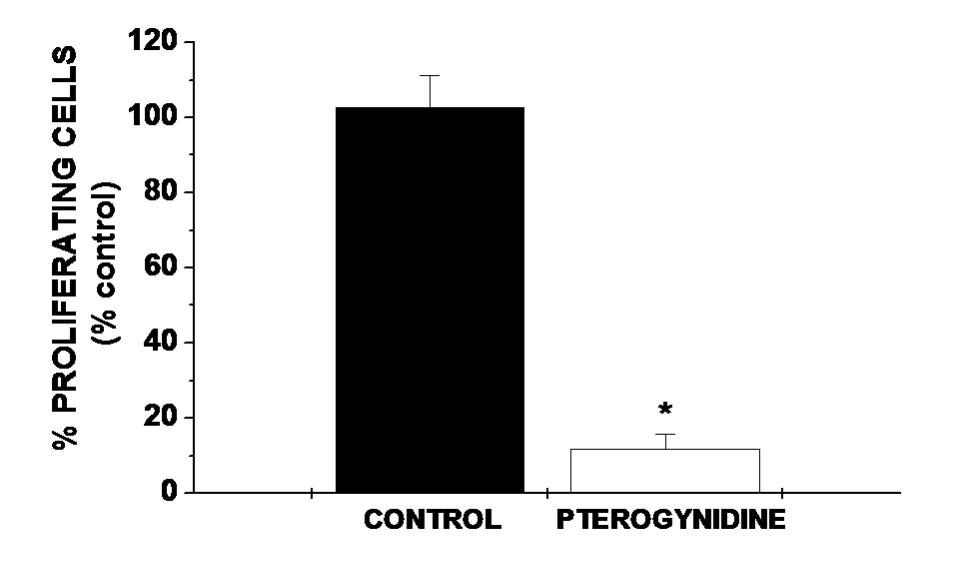
**Effect of Pt in HUVEC proliferation evaluated by BrdU assay**. Incubation with 8 μM Pt resulted in a very significant decrease in the percentage of proliferating cells. Bars represent the percentage of proliferating cells evaluated by the ratio of BrdU stained cells in 1000 hematoxylin stained nuclei. Assays were repeated five times and performed in triplicate. Statistical differences between groups were evaluated by one-way analysis of variance (ANOVA test) followed by the Dunnet test. *p < 0.01 vs Control.

**Figure 4:**
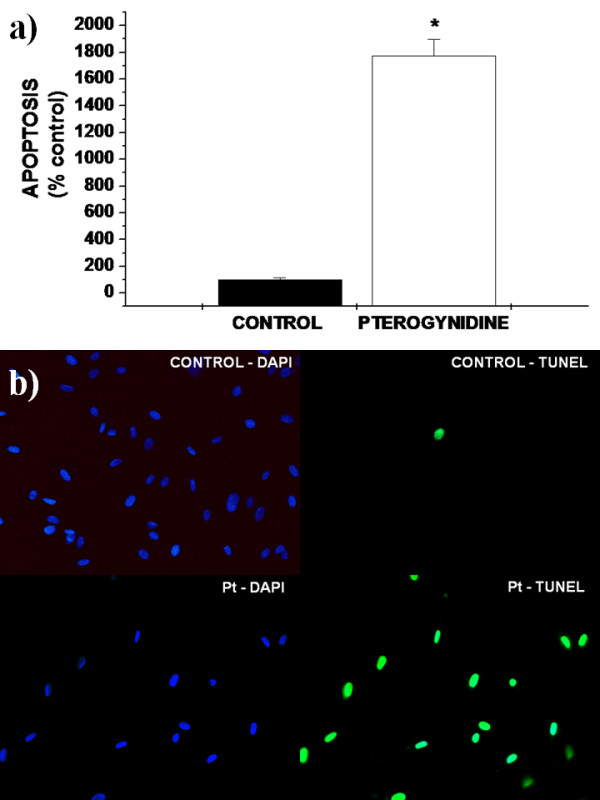
**Effect of Pt in HUVEC apoptosis evaluated by TUNEL assay**. a) The percentage of apoptotic cells was evaluated by the ratio between TUNEL-stained cells and DAPI-stained nuclei in every culture. Increased percentage of apoptotic cells was observed after incubation with 8 μM Pt. Results are expressed as percentage of control cells. Bars represent mean ± SD. Assays were repeated five times and performed in triplicate. Statistical differences between groups were evaluated by one-way analysis of variance (ANOVA test) followed by the Dunnet test. *p < 0.01 vs. Control. b) Apoptotic HUVEC representative photomicrographs stained with DAPI and detected by TUNEL assay after Pt treatment. Incubation with 8 μM Pt resulted in increased apoptosis determined as the percentage of positive cells per 1000 DAPI-stained nuclei. Glass coverslips were visualized under a fluorescence microscope (Nikon Eclipse 50i) at a magnification of 200×.

We observed a very significant decrease in the percentage of proliferating cells (11.79% ± 4.02%; *p < 0.01 vs. control) (Figure 3) and a strong increase in the percentage of apoptotic cells (1771.43% ± 122.81%; *p < 0.01 vs. control) (Figure 4a, Figure 4b) after incubation with 8 μM Pt as evaluated by BrdU and TUNEL assay, respectively.

**Figure 5:**
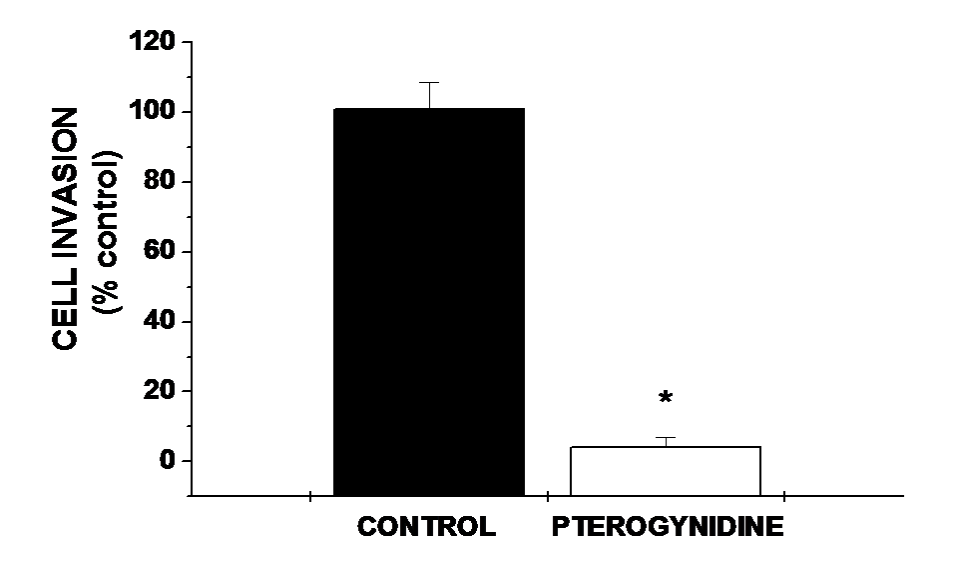
**Effect of Pt in HUVEC migration capacity evaluated by double chamber assay**. Incubation with 8 μM Pt resulted in decreased cell invasion. Bars represent the percentage of invading cells relative to the initial amount of cells cultured. Results are expressed as percentage of control cells. Assays were repeated five times and performed in triplicate. Statistical differences between groups were evaluated by one-way analysis of variance (ANOVA test) followed by the Dunnet test. *p < 0.01 vs. Control.

We next investigated whether Pt affected migration and invasion capacity of HUVEC using a double-chamber assay. As illustrated in Figure 5, Pt resulted in a very significant decrease in the migratory capacity of HUVEC (4.09% ± 2.95%; *p < 0.01 vs. control).

### Effects of Pt in cord-like structures formed by HUVEC

To form a new blood vessel, EC must assemble into vascular capillary structures. HUVEC are able to assemble into highly branched capillary-like structures when cultured on GFR-Matrigel. Therefore, we next examined whether Pt was able to affect the formation of these structures. Incubation of HUVEC with M199 medium and 0.1% DMSO (control), during 24 h, led to the formation of highly ramified cord-like structures. However, the presence of Pt led to a drastic reduction in the number of these cord-like structures into 0.2% ± 0.45% of control values (*p < 0.01 vs control).

**Figure 6:**
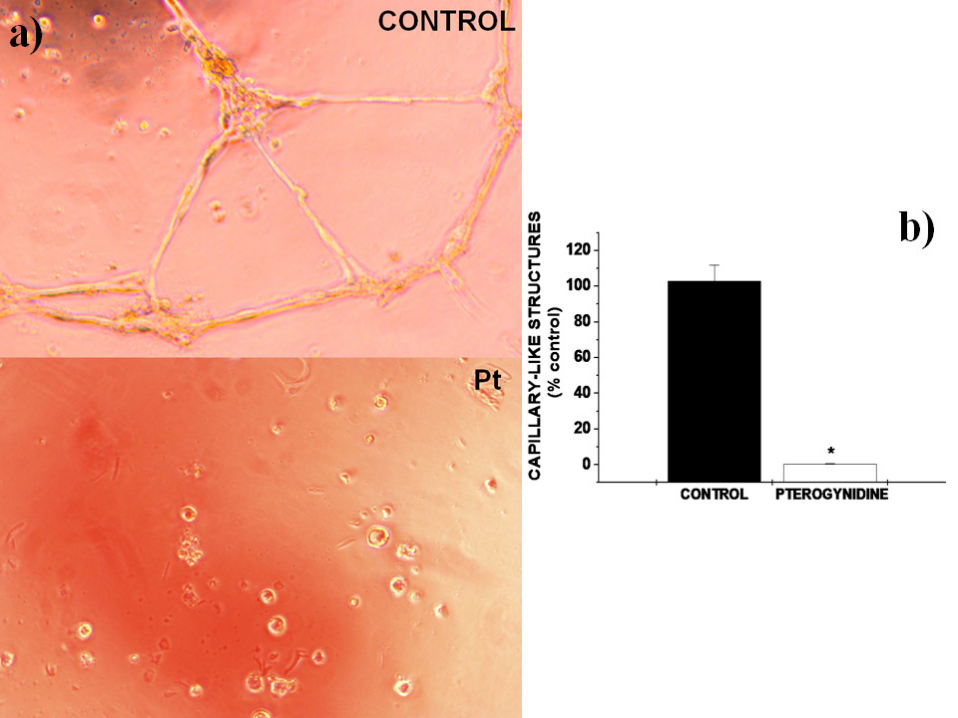
**Capillary-like structures in HUVEC cultures and semiquantification of the tube formation index**. a) Photomicrographs of capillary-like structures assembly in HUVEC cultures after treatment with Pt. In contrast to control cells, incubation with 8 μM Pt resulted in absence of ramifications with undifferentiated cells. Photomicrographs are representative of the whole cultures. Every culture was established in triplicate and visualized under an inverted microscope (×400 magnification). b) Semiquantification of the tube formation index in HUVEC after incubation with 8 μM Pt. There is a drastic reduction in the number of capillary-like structures formed upon incubation with this compound. Bars correspond to the percentage of the number of tubule-like structures comparatively to control. Statistical differences between groups were evaluated by one-way analysis of variance (ANOVA test) followed by the Dunnet test (mean ± SD; *p < 0.01 vs. Control).

Ramifications have pratically disappeared when HUVEC were treated with Pt in comparison to controls and just undifferentiated round-shaped cells could be seen under the microscope. These findings indicate that Pt at a concentration as low as 8 μM was able to inhibit vessel assembly, a crucial feature for the angiogenic process (Figure 6a and 6b).

### Pt inhibited NFκB activity in HUVEC

**Figure 7:**
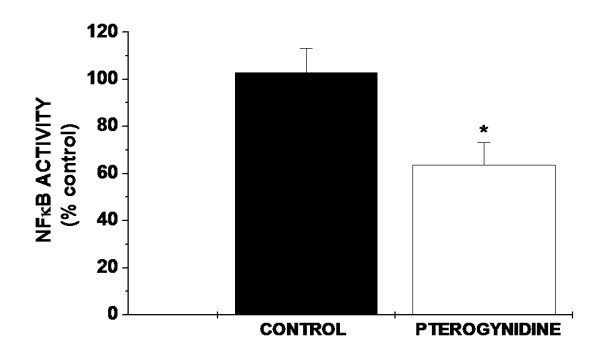
**Effects of Pt in NFκB p50 subunit activation in HUVEC**. A significant reduction in NFκB activity was found in HUVEC treated with 8 μM Pt in comparison to control. Results are mean ± SD of five independent experiments performed in triplicate. Statistical significance of difference between two groups was evaluated by one-way analysis of variance (ANOVA test) followed by the Dunnet test. (*p < 0.01 vs Control).

NFκB is a transcription factor involved in many cell fates, including cell growth, apoptosis, migration and stimulation of inflammatory factors [2,25]. The broad effects of 8 μM Pt in HUVEC prompted us to examine whether the activity of this factor was affected by the alkaloid using NFκB ELISA assay. A strong decrease in NFκB p50 subunit activity (63.67% ± 9.51%; *p < 0.01 vs. control) was found in HUVEC after incubation with Pt, indicating that NFκB signaling inactivation is one of the pathways triggered by Pt (Figure 7).

## Discussion

Angiogenesis has been described as one of the hallmarks of cancer, playing a fundamental role in tumor growth, invasion, and metastasis. In many pathological conditions, including chronic inflammation, diabetic retinopathy, rheumatoid arthritis or atherosclerosis, persistent upregulated angiogenesis is a common feature [26]. Thus, the central importance of angiogenesis and the understanding of how new blood vessels are formed have led to novel therapies designed to interrupt this process [27]. As part of our search for natural product-based antiangiogenic agents, we studied the effects of the pterogynidine alkaloid obtained from the Brazilian plant *A. glandulosa *in the angiogenic course, namely investigating cell viability, proliferation, migration, invasion and capillary-like structure formation using EC.

Natural products have been the most significant source of drugs and drug leads. Their dominant role in cancer chemotherapeutics is clear with about 74% of anticancer compounds being either natural products or natural product-derived [28]. Numerous bioactive chemical compounds of plant origin may influence the angiogenic activity of various cell types and may thus affect the formation of blood vessels [29,30]. Nowadays, several alkaloids are currently being exploited to target angiogenesis. Sanguinarine, an alkaloid isolated from the root of *Sanguinaria canadensis*, and Vinca alkaloids, obtained from *Catharanthus roseus *which are found in the rain forests of Madagascar, appear to be antiangiogenic natural products [31-34]. Moreover, halofuginone, an alkaloid from the plant *Dichroa febrifuga*, is considered to be a potent inhibitor of critical steps in angiogenesis progression [35].

The first experimental strategy for the screening of new anti-angiogenic compounds is the search of EC growth inhibitors [26]. Additionally, apoptosis appears to be fundamental in the initiation of the anti-angiogenic process [36]. In the current study, we demonstrated that Pt, a guanidine alkaloid, possesses antiangiogenic activity in several *in vitro *assays. Although cytotoxicity was only evaluated by MTT in the current study, incubation with 8 μM Pt led to a reduction in the number of viable cells only to 63% of control values (see MTT assay – Figure 2). Moreover, this decrease was significantly less than the ones obtained after treatment with the other higher Pt concentrations analyzed. These findings, together with the fact that 8 μM Pt was used in macrophage cultures in previous studies of our group with no significant cytotoxicity (unpublished data), prompt us to use the concentration of 8 μM for Pt during the whole study. Incubation with 8 μM Pt resulted in a drastic reduction in the percentage of EC proliferation and a strong increase of HUVEC apoptotic cells as compared to controls (Figure 3, Figure 4a and Figure 4b). Furthermore, 8 μM concentration of Pt was enough to induce an 8-fold decrease in the percentage of EC proliferation and a 17-fold increase in apoptotic rate as compared to controls. These results were expected, since antiproliferative and pro-apoptotic effects of other alkaloids and plant extracts have already been described in cancer cells [37,38], implying that a similar mechanism is probably occurring in EC as well.

Another plant from the Alchornea genus also possesses guanidine alkaloids.*Alchornea cordifolia *is one of the most widely-used plants in traditional medicine throughout Africa, mainly for its anti-inflammatory, antimicrobial and antiparasitic properties [39]. Two guanidine alkaloids, *N1, N2*-diisopentenyl guanidine and *N1, N2, N3*-triisopentenyl guanidine, isolated from *A. cordifolia *root bark, showed cytotoxic properties on cancer cells [40].

The alkaloids represent a very diverse group of medically significant compounds. They were originally defined as pharmacologically active, nitrogen-containing basic compounds of plant origin. Alkaloids are not unique to plants. They have also been isolated from numerous animal sources [41]. Guanidine alkaloids are present in marine organisms displaying various biological activities as cytotoxicity against tumor cell lines and inhibition of HIV-1 fusion [42-44].

EC invasive behaviour is another mainstay in angiogenesis. Invasive capacity requires extracellular matrix degradation and involves the activation of many EC signalling pathways. Our findings show a very effective decrease in Matrigel invasion in Pt-treated HUVEC, Pt being about 25 times more potent than control to inhibit the migratory capacity of HUVEC (Figure 5). Therefore, our findings suggest that Pt significantly reduced EC invasion and indicate the potential of this compound as an anti-angiogenic agent. In agreement with our study, Quesada et al. [26] pointed out that squalamine (Sq), a natural product isolated from the shark *Squalus acanthias*, inhibits angiogenesis and tumor growth, at least in part, by blocking mitogen-induced proliferation and migration of EC, thus preventing neovascularization of the tumor. Sq has a huge potential as a non-invasive anti-angiogenic therapy for ocular disorders, being evaluated in aged-related macular degeneration.

Finally, the anti-angiogenic activity of Pt was further demonstrated by tube formation assay performed on growth factor reduced-Matrigel. EC must assemble into capillary-like structures, in order to form a new blood vessel. We were able to show that Pt prevented the formation of these structures on Matrigel-coated plates, implying that EC differentiation into cord structures is also affected by this natural alkaloid. Most strikingly, network-like structures were not found when HUVEC was incubated with Pt and just isolated cells were observed under the inverted microscope, indicating that Pt can strongly disrupt tube formation (Figure 6a and 6b). Given the strong dependence of cancer in angiogenesis, our findings led us to propose that Pt can inhibit tumor growth through the suppression of blood vessel development, being a potential anti-tumor agent.

Vascular endothelial growth factor (VEGF) appears to play a pivotal role in the angiogenic process, being involved in a variety of normal and pathological circumstances [2,22,23]. Accordingly, VEGF induces vascular permeability, EC proliferation, migration and assembly into capillary structures. Given the fact that Pt abrogates all these processes in the current study, the anti-angiogenic action of this alkaloid might be due to the inhibition of VEGF signaling. Nevertheless, several other growth factors and signaling pathways play a role in angiogenesis. Similar to Pt, sinomenine (Sn), an alkaloid extracted from the Chinese medicinal plant *Sinomenium acutum *and used in rheumatoid arthritis treatment over 2000 years, also disrupts tube formation on Matrigel. Since tube formation of EC on the Matrigel depends on migration and morphological differentiation, mimmicking angiogenesis, Kok et al. [45] proposed that Sn inhibited basic fibroblast growth factor (bFGF)-induced angiogenesis. These authors further hypothesized that tube formation is highly dependent on cell-cell adhesion, implying a putative role for adhesion molecules such as ICAM-1 or E-selectin modulated by Sn in HUVEC.

These findings are in agreement with other studies in the literature, explaining the large effects of natural products in angiogenesis. A variety of herbs that are traditionally used for anticancer treatment also exhibit antiangiogenic activity through multiple interdependent processes (effects on gene expression, signal processing and enzyme activities), including *Artemisia annua*, *Viscum album*, *Curcuma longa *(curcumin), *Scutellaria baicalensis*, resveratrol and proanthocyanidin (grape seed extract), *Magnolia officinalis*, *Camellia sinensis *(green tea), *Ginkgo biloba*, quercetin, *Poria cocos, Zingiber officinalis (*ginger), *Panax ginseng*, *Rabdosia rubescens *and Chinese destagnation herbs. A potential advantage of phytochemicals and other compounds derived from natural health products is that they may act through multiple cell-signalling pathways and reduce the development of cancer cell resistance [46]. Although the studies showed the regulation of multiple signalling pathways by natural products, the evaluation of possible mechanisms by which Pt act were still not well understood.

Finally, we decided to explore whether Pt could block the transcription factor NFκB, which generally enhances cell proliferation and survival by targeting multiple sites of the pathway. NFκB plays an important role in carcinogenesis, as well as in the regulation of immune and inflammatory responses, inducing the expression of diverse target genes that promote cell proliferation, regulate apoptosis, facilitate angiogenesis and stimulate invasion and metastasis. In addition, many cancer cells show aberrant or constitutive NFκB activation which mediates resistance to chemotherapy and radiotherapy [47]. Therefore, given the critical role of NFκB in the pathogenesis of cancer, specific inhibitors against this factor would be useful in cancer therapy. Agents that prevent cancer or inflammation have been found to suppress NFκB activation. Numerous reports indicate that ancient plants and their components are potent as NFκB inibitors [48].

In the present research, Pt inhibited endothelial NFκB activity (Figure 7). NFκB was previously shown to be down-regulated by various chemopreventive phytochemicals [49,50]. Curcumin is a phytochemical that possesses anti-angiogenic properties. It decreases migration and invasion of EC and down-regulates NFκB and inhibits IκB kinase, thereby suppressing proliferation and inducing apoptosis [51]. According to Mohan et al. [52], NFκB specific inhibitors as parthenolide, inhibit EC proliferation as well as NFκB DNA binding, exerting anti-endothelial cell sprouting activity. Accordingly, and although further studies are required, the current results suggests that inactivation of NFκB by the alkaloid is probably one of the mechanisms involved in the observed anti-angiogenic effects on HUVEC.

Recently, it has been reported that many natural products can act as angiogenesis inhibitors. To our knowledge, this was the first report concerning the effects of Pt in angiogenesis using HUVEC. Mojzis et al. [53] propose that angiogenesis inhibitors are likely to change the face of medicine in the next decade and particularly the chemopreventive agents that act on the highly specialized biology of EC during neovascularization deserve special attention.

## Conclusion

Taken together, representing the sequential events in the angiogenic process, Pt treatment showed strong anti-angiogenic effects possibly through the inhibition of EC proliferation, invasion and assembly into capillary-like structures together with apoptosis induction. Our findings also identify the mode of action of this natural product through the inhibition of NFκB. Despite the fact that cytotoxic effects were not addressed in the current study for 8 μM concentration of Pt, these results demonstrate that Pt might be a novel inhibitor of angiogenesis, suggesting that it may be important in the development of pharmaceutical drugs or providing model molecules for the discovery of potential new agents useful in angiogenesis dependent diseases, especially tumor treatment or prevention. Nevertheless, *in vivo *studies are mandatory in order to support these findings.

## Abbreviations

EC: Endothelial Cells; Pt: Pterogynidine; HUVEC: Human Umbilical Vein Endothelial; NFκB: Cells Nuclear Factor κB; ANOVA: One-Way Analysis of Variance; BrdU: bromodeoxyuridine; TUNEL: Terminal Deoxynucleotidyl Transferase Mediated Deoxyuridine Triphosphate Nick-End Labeling; GFR-Matrigel: Growth Factor Reduced-Matrigel; FBS: Fetal Bovine Serum; ECGS: Endothelial Cell Growth Supplement; DMSO: Dimethyl Sulfoxide; MTT: [3-(4,5-dimethylthiazol-2-yl)-2,5-diphenyl tetrazolium bromide].

## Competing interests

The authors declare that they have no competing interests.

## Authors' contributions

FCML performed the assays. AR and AP helped in some experiments and acquisition of data. LOR, DHSS and VSB colaborated in phytochemical procedures. FCML, IZC and RS designed and coordenated the study and also helped to draft the manuscript. IZC and IA have been involved in revising the manuscript critically for important intellectual content. All authors have read and approved the final manuscript.

## Pre-publication history

The pre-publication history for this paper can be accessed here:


